# A Simple Substitute for “Eiweissmilch.”

**Published:** 1914-08

**Authors:** 


**A Simple Substitute for "Eiweissmilch."** Stoeltzner in Pediatrics, May, 1914, recommends his Larosan, a casein-calcium powder, which is tasteless and soluble in hot milk, to take the place of Finkelstein's albumin-milk. He has used it in 66 cases and found it at least equal to albumin-milk. The number of cases in which he has tried this food is far too small to form any opinion of its value and others, beside the originator, should be heard from before endorsing his claims.—C. G. L-W.

